# Draft Genome Sequence of *Burkholderia stabilis* LA20W, a Trehalose Producer That Uses Levulinic Acid as a Substrate

**DOI:** 10.1128/genomeA.00795-16

**Published:** 2016-08-04

**Authors:** Yuya Sato, Hideaki Koike, Susumu Kondo, Tomoyuki Hori, Manabu Kanno, Nobutada Kimura, Tomotake Morita, Kohtaro Kirimura, Hiroshi Habe

**Affiliations:** a Environmental Management Research Institute, National Institute of Advanced Industrial Science and Technology (AIST), Tsukuba, Ibaraki, Japan; b Bioproduction Research Institute, AIST, Tsukuba, Ibaraki, Japan; c Research Institute for Sustainable Chemistry, AIST, Tsukuba, Ibaraki, Japan; d Department of Applied Chemistry, Faculty of Science and Engineering, Waseda University, Tokyo, Japan

## Abstract

*Burkholderia stabilis* LA20W produces trehalose using levulinic acid (LA) as a substrate. Here, we report the 7.97-Mb draft genome sequence of *B. stabilis* LA20W, which will be useful in investigations of the enzymes involved in LA metabolism and the mechanism of LA-induced trehalose production.

## GENOME ANNOUNCEMENT

Cellulose, the most abundant nonedible biomass resource, is important for the synthesis of many bio-based chemicals (1). Of the diverse chemicals other than sugars that can be synthesized from cellulose, levulinic acid (LA) is a promising building block for chemical production. In fact, it is considered one of the top 12 building blocks by the U.S. Department of Energy (2). LA can be converted into a variety of useful compounds via chemical processes (3). However, few reports have focused on the microbial conversion of LA into such chemicals, because short-chain organic acids, such as LA, can inhibit microbial growth at moderate concentrations (4). Recently, we isolated and identified LA-utilizing bacteria (5, 6), including *Burkholderia stabilis* LA20W. The most notable feature of the *B. stabilis* LA20W strain is that it produces trehalose extracellularly in the presence of LA but not glucose (5). However, the molecular basis of trehalose production with LA is unclear.

The draft genome of *B. stabilis* LA20W was generated using the next-generation sequencing platform MiSeq (Illumina, San Diego, CA, USA). Both paired-end and mate-pair DNA libraries (insert size, ~500 bp) were prepared using the NEBNext Ultra DNA library prep kit for Illumina (New England BioLabs, Ipswich, MA, USA) and the Nextera mate-pair sample prep kit (Illumina), respectively, and sequenced on the Illumina MiSeq system using the MiSeq reagent kit, version 2 (Illumina). The resulting sequencing data included 3.51-Mb paired-end and 5.51-Mb mate-pair reads, with average read lengths of approximately 250 and 2,000 bp, respectively. Genomic sequence assembly using ALLPATHS-LG version 46449 (7) produced four scaffolds composed of 23 contigs and a 7.97-Mb draft genome sequence at a 166-fold coverage from both the paired-end and mate-pair libraries. The length of the longest scaffold was 3,620,542 bp, and the *N*_50_ length was 3,210,539 bp for two scaffolds. A total of 7,548 protein-coding genes were predicted using Glimmer 3.02 (8) and annotated using NCBI-BLAST-2.2.29 (BLASTP) with RefSeq version 65 (9, 10). A total of 56 tRNA-encoding and 10 rRNA-encoding genes were also identified by tRNAscan-SE-1.3.1 and RNAmmer-1.2, respectively (11, 12).

Trehalose accumulation is a common cell defense strategy against a variety of stressful conditions (13, 14). In the draft genome of *B. stabilis* LA20W, genes involved in *de novo* trehalose biosynthesis were identified. The OtsA-OtsB pathway is the most common mode of *de novo* trehalose biosynthesis, in which the trehalose-6-phosphate synthase OtsA catalyzes the transfer of the nucleoside diphosphate-activated glucose UDP-glucose to glucose-6-phosphate to yield trehalose-6-phosphate; subsequently, the trehalose-6-phosphate phosphatase OtsB dephosphorylates trehalose-6-phosphate to yield trehalose (15). At least three *otsA* gene homologues and one *otsB* gene were annotated in the draft genome. The TreY-TreZ pathway is another mode of trehalose production. We identified a *treZ* gene, which encodes maltooligosyl trehalose trehalohydrolase (TreZ). Transcriptomic analyses based on the draft genome sequence may provide novel insights into the LA-induced extracellular production of trehalose by *B. stabilis* LA20W.

### Nucleotide sequence accession numbers.

The *B. stabilis* LA20W draft genome sequence has been deposited as 23 contigs (accession numbers BDCP01000001 to BDCP01000023) and four scaffolds (accession numbers DF978417 to DF978420) in DDBJ/EMBL/GenBank. The version described in this paper is the first version.
